# Disseminated pulmonary emboli caused by mercury: A rare consequence of Munchausen's syndrome

**DOI:** 10.1002/ccr3.3435

**Published:** 2020-10-22

**Authors:** Wen Shi, Yang Jiao

**Affiliations:** ^1^ Department of Gastroenterology Peking Union Medical College Hospital Chinese Academy of Medical Sciences & Peking Union Medical College Beijing China; ^2^ Department of General Internal Medicine Peking Union Medical College Hospital Chinese Academy of Medical Sciences & Peking Union Medical College Beijing China

**Keywords:** factitious disorde, mercury poisoning, Munchausen's syndrome, pulmonary embolism

## Abstract

To learn about the imaging characteristics of pulmonary emboli caused by metallic mercury and be aware that an underlying psychiatric condition might exist when mercury poisoning is not caused by occupational exposure.

## Graphical abstract

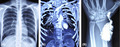

A 25‐year‐old asymptomatic woman was referred with diffuse, high‐density opacities in a chest radiograph (Figure 1A) and disseminated, metallic dots in the lung parenchyma (Figure 1B, arrows) on 3D‐reconstructed CT. Her oxygen saturation was 98%. These unusual appearances prompted serum calcium (normal) and heavy metal testing; serum mercury levels were raised at 13.5 ng/mL (normal: <4 ng/mL). Disseminated mercury in the lungs was diagnosed, but the origin of the mercury remained uncertain. The patient denied self‐harm or mercury exposure. However, on further questioning, her family revealed that she had received previous surgery to remove mercury from her left arm 2 years previously, with an X‐ray at that time showing high‐density soft tissue opacities in her left forearm consistent with mercury self‐injection (Figure 1C). There were no obvious financial or emotional rewards from self‐harming, and depression and acute psychosis were excluded. Munchausen's syndrome (factitious disorder) was suspected, with disseminated mercury deposition apparently an unintended consequence of fabricating illness through peripheral mercury injection. She was referred to the Psychology Department while receiving chelation therapy. Regrettably, she was lost to follow‐up after 3 months, which is not unusual for patients with Munchausen's syndrome.

**FIGURE 1:**
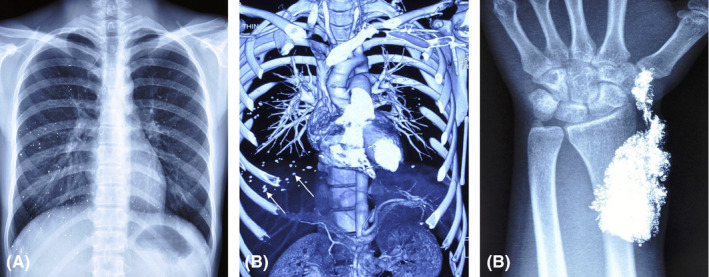
(A) Chest radiograph showing diffuse, high‐density opacities distributed in both lungs. (B) 3D‐reconstructed chest CT demonstrating disseminated metallic dots in the lung parenchyma (arrows). (C) Previous radiograph of the left forearm showing high‐density soft tissue opacities in the soft tissues

Munchausen's syndrome is a factitious disorder imposed on the self and usually difficult to identify due to deceptive misrepresentation. Patients intentionally fake or cause symptoms of an illness and/or injury in themselves, even in the absence of obvious external rewards.^1^ Self‐injection of mercury has been reported as a rare complication of psychiatric diseases, including Munchausen's syndrome.^2^ Exposure to elemental mercury is usually by inhalation of its vapor, and it is poorly absorbed via intact skin. In our case, the patient subcutaneously injected mercury,however, even after surgical clearance, the residue at injection sites still permeated into the circulation and led to mercury embolization to the lungs, which she did not expect and revealed the underlying diagnosis.

## CONFLICTS OF INTEREST

None declared.

## AUTHOR CONTRIBUTIONS

WS: wrote the initial draft of the manuscript. YJ: critically appraised and revised the overall content of the manuscript. Both authors: read and approved the final manuscript.

## ETHICAL APPROVAL

Our study has been granted an exemption from the review by the Institutional Review Board of Peking Union Medical College Hospital.

## CONSENT FOR PUBLICATION

Written informed consent was obtained from the patient's brother (authorized legal representative) for publication of this case report and any accompanying images.
